# Effect of Fear of Falling on Turning Performance in Parkinson’s Disease in the Lab and at Home

**DOI:** 10.3389/fnagi.2018.00078

**Published:** 2018-03-27

**Authors:** Linda Haertner, Morad Elshehabi, Laura Zaunbrecher, Minh H. Pham, Corina Maetzler, Janet M. T. van Uem, Markus A. Hobert, Svenja Hucker, Susanne Nussbaum, Daniela Berg, Inga Liepelt-Scarfone, Walter Maetzler

**Affiliations:** ^1^Center for Neurology and Hertie-Institute for Clinical Brain Research, Department of Neurodegeneration, University of Tübingen, Tübingen, Germany; ^2^German Center for Neurodegenerative Diseases, Tübingen, Germany; ^3^Department of Neurology, Universitätsklinikum Schleswig-Holstein, Christian-Albrechts-Universität zu Kiel, Kiel, Germany

**Keywords:** home assessment, inertial measurement unit, Parkinson’s disease, quantitative assessment, turning

## Abstract

**Background:** Parkinson’s disease (PD) is a neurodegenerative movement disorder associated with gait and balance problems and a substantially increased risk of falling. Falls occur often during complex movements, such as turns. Both fear of falling (FOF) and previous falls are relevant risk factors for future falls. Based on recent studies indicating that lab-based and home assessment of similar movements show different results, we hypothesized that FOF and a positive fall history would influence the quantitative turning parameters differently in the laboratory and home.

**Methods:** Fifty-five PD patients (43 underwent a standardized lab assessment; 40 were assessed over a mean of 12 days at home with approximately 10,000 turns per participant; and 28 contributed to both assessments) were classified regarding FOF and previous falls as “vigorous” (no FOF, negative fall history), “anxious” (FOF, negative fall history), “stoic” (no FOF, positive fall history) and “aware” (FOF, positive fall history). During the assessments, each participant wore a sensor on the lower back.

**Results:** In the lab assessment, FOF was associated with a longer turning duration and lowered maximum and middle angular velocities of turns. In the home evaluations, a *lack* of FOF was associated with lowered maximum and average angular velocities of turns. Positive falls history was not significantly associated with turning parameters, neither in the lab nor in the home.

**Conclusion:** FOF but not a positive fall history influences turning metrics in PD patients in both supervised and unsupervised environments, and this association is different between lab and home assessments. Our findings underline the relevance of comprehensive assessments including home-based data collection strategies for fall risk evaluation.

## Introduction

Up to 68% of patients with Parkinson’s disease (PD) fall at least once a year (Canning et al., 2009; Voss et al., 2012; Fasano et al., 2017). Falls reduce patient quality of life and could lead to severe injuries, such as hip fractures (Voss et al., 2012; Yang et al., 2016). Deficits in turning performance contribute relevantly to the occurrence and frequency of falls in patients with this disease (King et al., 2012; Yang et al., 2016). This association between turning deficits and fall frequency is most likely due to the complexity of the movement. Turning requires inter-limb coordination, complex coupling between posture and gait, and continuous, dynamic movement of the individual’s center of gravity (Cheng et al., 2014; Mellone et al., 2016). Recent studies performed in a supervised environment show that PD patients tend to turn slower, with more steps and a reduced turning angle than controls do (El-Gohary et al., 2013; Cheng et al., 2014; Mellone et al., 2016). Moreover, PD patients show a simultaneous rotation of the head, thorax, and pelvis during turning (they move “en bloc”) (Crenna et al., 2007; Mellone et al., 2016; Yang et al., 2016). This axial rigidity may further increase lateral instability during this movement. A narrower base of support which leads to an external, unbalanced center of gravity during the turn, resulting in a higher risk of falling, may also contribute to an increased fall risk during turning in this population (Mellone et al., 2016).

Recent studies have indicated that fear of falling (FOF) (Allen et al., 2013; Fasano and Bloem, 2013; Bryant et al., 2014; Almeida et al., 2015) and a positive history of falls (Bloem et al., 2001; Pickering et al., 2007; Voss et al., 2012; Allen et al., 2013; Bryant et al., 2014) contribute to future falls in PD patients. The influence of FOF on future falls has also been demonstrated in non-PD cohorts, including cohorts with a poor balance status and reduced mobility function (Mak and Pang, 2009; Delbaere et al., 2010a). In this context, it is relevant to note that PD patients clearly have higher FOF scores than older adults without PD (Pham et al., 2017).

Based on this information, we evaluated the impact of FOF and a history of falls on quantitative parameters of turning in both supervised and unconstrained environments. We included both environments due to the following reasons: (i) assessment performed in the habitual environment may reflect the person’s usual performance and may have a higher relevance for our understanding of the health-related quality of life of a patient than assessments performed in a doctor’s office or a scientific laboratory (El-Gohary et al., 2013; Giannouli et al., 2016; van Uem et al., 2016; Zhang et al., 2017), and (ii) the use of a novel technique allows the collection of the activity patterns of PD patients in the home environment with validated algorithms (El-Gohary et al., 2013; Mancini et al., 2015; Pham et al., 2017). There is increasing evidence that parameters of similar activities, assessed in one person first in the laboratory and then in the home environment may substantially differ (Toosizadeh et al., 2015; Giannouli et al., 2016; Zhang et al., 2017). We hypothesized that both factors, FOF and a history of falls, independently affect turning metrics in both environments.

## Materials and Methods

### Study Cohort Demographics and Clinical Assessments

The ABC-PD study (amyloid-beta in cerebrospinal fluid as a risk factor for cognitive dysfunction in Parkinson’s disease) was approved by the ethics committee of the Medical Faculty of the University of Tübingen (protocol no. 686/2013BO1) and was performed in accordance with the ethical standards outlined in the 1964 Declaration of Helsinki and its later amendments.

We obtained informed consent from 55 PD patients prior to enrolment. All of the patients fulfilled the following inclusion criteria: 50–85 years of age, diagnosis of PD according to the UK Brain-Bank criteria, and an ability to communicate well with the investigator and to understand and comply with the requirements of the study. Dementia served as an exclusion criterion (Emre et al., 2007). Forty-three subjects (31 males and 12 females, mean age: 67; **Table 1**) were included in the lab-based substudy, and 40 subjects (29 males and 11 females, mean age: 67; **Table 2**) were included in the home-based evaluation. Twenty-eight of the participants contributed to both substudies, which allowed us to intra-individually compare lab-collected turning parameters with those collected at home (**Table 3**).

**Table 1:** Demographic, clinical, and iTUG data in the lab assessment.

	“The vigorous” FOF-	“The anxious” FOF+	“The stoic” FOF-	“The aware” FOF+	*p*-value	Cohort
	Previous falls-	Previous falls-	Previous falls+	Previous falls+		
*N* (female)	18 (5)	12 (3)	8 (3)	5 (1)	0.86	43 (12)
Age (years)	65 ± 10	64 ± 19	68 ± 9	67 ± 6	0.85	65 ± 12
UPDRS III^1^ (0–132)	21 (10–34)	31 (15–56) ^∗^	23 (18–39) ^(‡)^	30 (26–42) ^(∗)^	**0.003**	27 (10–56)
H&Y (1–5)	4/13/1/0/0	0/11/1/0/0	2/6/0/0/0	2/1/2/0/0	0.05	8/31/4/0/0
MMSE^1^ (0–30)	28 (25–30)	28 (24–30)	29 (24–30)	29 (27–30)	0.57	28 (24–30)
FES-I^1^ (0–64)	17 (16–19)	23 (20–35) ^∗^	17 (16–19) ^‡^	27 (21–29) ^∗ #^	**0.0001**	18 (16–35)
Disease duration^1^ (years)	5 (2–15)	6 (3–13)	4 (3–5)	5 (2–10)	0.18	5 (2–15)
Freezing of gait (yes vs. no) (Vogler et al., 2015)	y:4, n:14	y:5, n:7	y:2, n:6	y:2, n:3	0.38	y:13, n:30
Levodopa equivalent dose^1^ (mg)	415 (100–1055)	650 (220–1550)	525 (180–756)	457 (400–980)	0.19	460 (100–1550)
CSF-amyloid-beta_1-42_ (normal vs. lowered) (Palmqvist et al., 2014)	n:10, l:8	n:5, l:7	n:2, l:6	n:2, l:3	0.53	n:19, l:24
**iTUG turn 1**						
Turn duration (s)	2.34 ± 0.53	2.85 ± 0.67	2.25 ± 0.56	3.44 ± 1.09 ^∗ #^	**0.02**	2.59 ± 0.74
Turn angle (°)	183 ± 7	178 ± 9	181 ± 4	172 ± 12	0.16	180 ± 8
Average angular velocity (°/s)	81 ± 18	71 ± 15	78 ± 16	58 ± 13	0.20	75 ± 17
Maximum angular velocity (°/s)	157 ± 33	133 ± 28	163 ± 24	117 ± 35	0.07	147 ± 33
Start angular velocity (°/s)	29 ± 20	23 ± 14	19 ± 10	32 ± 13	0.43	26 ± 16
Middle angular velocity (°/s)	123 ± 32	122 ± 38	143 ± 24	81 ± 28 ^(‡) #^	**0.02**	122 ± 35
End angular velocity (°/s)	24 ± 11	17 ± 7	21 ± 7	23 ± 12	0.72	21 ± 10
**iTUG turn 2**						
Turn duration (s)	4.15 ± 0.56	4.94 ± 0.72	4.41 ± 0.81	6.07 ± 1.79 ^∗ (‡) #^	**0.002**	4.64 ± 1.03
Turn angle (°)	180 ± 7	174 ± 11	177 ± 6	177 ± 4	0.88	177 ± 8
Average angular velocity (°/s)	72 ± 16	61 ± 15	71 ± 16	50 ± 14	0.22	66 ± 17
Maximum angular velocity (°/s)	152 ± 43	115 ± 22 ^(∗)^	147 ± 17	95 ± 17 ^∗ (#)^	**0.03**	134 ± 37
Start angular velocity (°/s)	26 ± 17	22 ± 13	24 ± 8	19 ± 14	0.97	24 ± 14
Middle angular velocity (°/s)	116 ± 32	91 ± 31	95 ± 44	70 ± 18	0.26	100 ± 36
End angular velocity (°/s)	8 ± 3	9 ± 4	12 ± 7	8 ± 5	0.29	9 ± 5

*Calculations were performed with ANOVA and a *post hoc* Student’s *t*-tests (demographic and clinical data), and with a linear regression model corrected for gender, and UPDRS III (iTUG data). In case of non-normally distributed data^*1*^, data are presented with median and range, and calculations were performed with the Kruskal–Wallis and *post hoc* Wilcoxon tests. Statistically significant differences are marked in bold. *Post hoc* analyses were Bonferroni corrected (*p* < 0.05/4 = 0.0125). *Post hoc* values 0.0125 ≤*p*<0.05 are presented in brackets. ^∗^ Against “the vigorous”; ^‡^ against “the anxious”; ^#^ against “the stoic.” CSF, cerebrospinal fluid; FES-I, The Falls Efficacy Scale International; FOF, fear of falling; H&Y, Hoehn and Yahr stage; MMSE, Mini-Mental-State-Examination; iTUG, instrumented Timed up and Go Test; UPDRS III, Unified Parkinson’s Disease Rating Scale.*

**Table 2:** Demographic, clinical, and quantitative turning data in the home assessment.

	“The vigorous” FOF-	“The anxious” FOF+	“The stoic” FOF-	“The aware” FOF+	*p*-value	Cohort
	Previous falls-	Previous falls-	Previous falls+	Previous falls+		
*N* (female)	19 (5)	10 (2)	6 (2)	5 (2)	0.48	40 (11)
Age (years)	66 ± 9	65 ± 6	68 ± 8	66 ± 8	0.95	66 ± 8
UPDRS III^1^ (0–132)	21 (10–38)	29 (15–56)	23 (15-30)	29 (15-30)	0.06	23 (10-56)
H&Y (1–5)	2/15/2/0/0	9/1/0/0/0	2/4/0/0/0	2/1/2/0/0	0.07	6/29/5/0/0
MMSE^1^ (0–30)	28 (25–30)	28 (24–30)	29 (24–30)	28 (26–30)	0.51	28 (24–30)
FES-I^1^ (0–64)	16 (16–19)	23 (20–35) ^∗^	17 (16–19) ^‡^	27 (25–41) ^∗ ‡#^	**0.0001**	18 (16–41)
Disease duration^1^ (years)	4 (2–15)	6 (3–13)	3 (2–5)	5 (2–12)	0.13	4 (2–15)
Freezing of gait (yes/no) (Vogler et al., 2015)	y:4, n:15	y:4, n:6	y:2, n:4	y:2, n:3	0.64	y:12, n:28
Levodopa equivalent dose^1^ (mg)	415 (100–1055)	735 (220–1252)	460 (100–715)	429 (400–1338)	0.11	457 (100–1338)
CSF amyloid-beta_1-42_ (normal vs. lowered) (Palmqvist et al., 2014)	n:9, l:10	n:2, l:8	n:2, l:4	n:4, l:1	0.14	n:17, l:23
Duration of measurement (days)^1^	12 ± 2	12 ± 2	12 ± 2	13 ± 1	0.63	12 ± 2
Number of turns/day^1^	787 ± 366	912 ± 635	770 ± 299	710 ± 341	0.60	804 ± 417
Turn angle (°)	122 ± 3	121 ± 3	119 ± 2	118 ± 5	0.11	121 ± 3
Turn duration (s)	2.47 ± 0.23	2.49 ± 0.19	2.71 ± 0.26	2.38 ± 0.33	0.07	2.50 ± 0.25
Average angular velocity (°/s)	59 ± 6	59 ± 5	52 ± 6 ^(∗) (‡)^	61 ± 8 ^#^	**0.04**	58 ± 6
Maximum angular velocity (°/s)	183 ± 18	185 ± 9	167 ± 14 ^(∗) (‡)^	194 ± 20 ^#^	**0.04**	182 ± 17
Start angular velocity (°/s)	24 ± 6	23 ± 4	20 ± 5	25 ± 6	0.34	23 ± 5
Middle angular velocity (°/s)	73 ± 13	77 ± 6	68 ± 6	79 ± 10	0.23	74 ± 11
End angular velocity (°/s)	25 ± 5	25 ± 4	22 ± 4	28 ± 5	0.33	25 ± 5

*Calculations were performed with ANOVA and a *post hoc* Student’s *t*-tests (demographic and clinical data), and with a linear regression model corrected for gender, and UPDRS III (quantitative turning data). In case of non-normally distributed data^*1*^, data are presented with median and range and calculations were performed with the Kruskal–Wallis and *post hoc* Wilcoxon tests. Statistically significant differences are marked in bold. *Post hoc* analyses were Bonferroni corrected (*p* < 0.05/4 = 0.0125). *Post hoc* values 0.0125 ≤*p* < 0.05 are presented in brackets. ^∗^ Against “the vigorous”; ^‡^ against “the anxious”; ^#^ against “the stoic.” CSF, cerebrospinal fluid; FES-I, The Falls Efficacy Scale International; FOF, fear of falling; H&Y, Hoehn and Yahr stage; MMSE, Mini-Mental-State-Examination; iTUG, instrumented Timed up and Go Test; UPDRS III, Unified Parkinson’s Disease Rating Scale.*

**Table 3:** Ratio of turning parameters in the home versus the lab assessment.

	“The vigorous” FOF-	“The anxious” FOF+	“The stoic” FOF-	“The aware” FOF+	*p*-value	Cohort
	Previous falls-	Previous falls-	Previous falls+	Previous falls+		
*N* (female)	13 (4)	7 (1)	5 (2)	3 (0)	0.52	28 (7)
Age (years)	65 ± 10	65 ± 7	67 ± 8	64 ± 10	0.98	65 ± 9
UPDRS III^1^ (0–132)	21 (10–33)	28 (15–37)	25 (19–30)	29 (26–30)	0.09	23 (10–37)
H&Y (1–5)	1/11/1/0/0	0/1/6/0/0	1/4/0/0/0	2/0/1/0/0	0.50	4/21/3/0/0
MMSE^1^ (0–30)	28 (25–30)	28 (27–30)	29 (24–30)	30 (27–30)	0.74	29 (24–30)
FES-I^1^ (0–64)	17 (16–18)	22 (20–35)^∗^	17 (16–19)^‡^	28 (27–29)^∗#^	**0.0001**	18 (16–35)
Disease duration^1^ (years)	5 (2–15)	5 (3–13)	3 (2–5)	4 (2–5)	0.23	4 (2–15)
Freezing of gait (yes/no) (Vogler et al., 2015)	y:3, n:10	y:2, n:5	y:2, n:3	y:1, n:2	0.98	y:8, n:20
Levodopa equivalent dose^1^ (mg)	415 (100–1055)	690 (220–1252)	500 (375–715)	420 (400–457)	0.24	460 (100–1252)
CSF amyloid-beta_1-42_ (normal vs. lowered) (Palmqvist et al., 2014)	n:8, l:5	n:2, l:5	n:2, l:3	n:2, l:1	0.47	n:14, l:14
Turn duration (ratio)	0.75 ± 0.12	0.71 ± 0.06	0.82 ± 0.14	0.63 ± 0.10	0.07	0.74 ± 0.12
Average angular velocity (ratio)	0.77 ± 0.11	0.80 ± 0.12	0.71 ± 0.14	0.95 ± 0.02	0.07	0.79 ± 0.13
Maximum angular velocity (ratio)	1.25 ± 0.19	1.33 ± 0.22	1.11 ± 0.16	1.58 ± 0.04 ^(∗) #^	**0.036**	1.28 ± 0.22
Start angular velocity (ratio)	1.03 ± 0.49	1.12 ± 0.76	0.85 ± 0.32	0.85 ± 0.21	0.70	1.00 ± 0.51
Middle angular velocity (ratio)	0.63 ± 0.13	0.67 ± 0.13	0.60 ± 0.15	0.95 ± 0.25 ^∗ (‡) #^	**0.027**	0.67 ± 0.17
End angular velocity (ratio)	1.64 ± 0.53	1.61 ± 0.34	1.47 ± 0.35	1.55 ± 0.53	0.92	1.59 ± 0.44

*Calculations of the ratios of turns performed in the lab [mean of two turns as performed during the instrumented Timed up and Go (iTUG) test, see text for details] and at home (i.e., home parameter divided by respective lab parameter) were performed with a linear regression model corrected for gender and UPDRS III. Statistically significant differences are marked in bold. *Post hoc* analyses were Bonferroni corrected (*p* < 0.05/4 = 0.0125). *Post hoc* values 0.0125 ≤*p* < 0.05 are presented in brackets. ^∗^ Against “the vigorous”; ^‡^ against “the anxious”; ^#^ against “the stoic.”*

Patients in the lab-based assessment were measured during their medication ON phase, i.e., during the study participant’s perception of having a good “on” phase after regular intake of medication (Elshehabi et al., 2016).

All participants were assessed with the motor part of the Unified Parkinson’s Disease Rating Scale (MRS-UPDRS-III) (Goetz et al., 2008), the Hoehn and Yahr (H&Y) Scale (Hoehn and Yahr, 1967), the Mini-Mental-State-Examination (MMSE) (Folstein et al., 1975), and the Freezing of Gait Questionnaire (Vogler et al., 2015). FOF was assessed with the German version of the Falls Efficacy Scale-International (FES-I) (Yardley et al., 2005). Fall history was assessed with a semi-structured interview by asking the participants about occurrence and frequency of falls during the last 12 months (Heinzel et al., 2016). A FES-I score of 19 (cut-off between low and moderate/high FOF cut-point) (Delbaere et al., 2010b), and two falls in the preceding year served as cut off values. All participants also underwent lumbar puncture and cerebrospinal fluid amyloid-beta_1-42_ was evaluated.

### Turning Assessment in the Lab

In this substudy, PD patients performed two instrumented Timed up and Go (iTUG) trials over a walking distance of 7 m at convenient gait pace. The iTUG includes two 180° turns: one during walking, and one when sitting down again. Data was assessed with a Mobility Lab^®^ inertial measurement unit (IMU) at the lower back (OPAL system, APDM, Inc., Portland, OR, United States).

### Turning Assessment at Home

In this substudy, PD patients continuously wore a small IMU (RehaGait^®^, Hasomed, Magdeburg) in an elastic belt at the lower back for a median of 12 days. The feasibility and usability of this procedure have recently been demonstrated in an independent PD cohort (Ferreira et al., 2015; van Uem et al., 2016).

### Data Extraction

For the detection of turns and the extraction of turn metrics in both substudies, we used a recently published algorithm developed by our group (Pham et al., 2017). The turn metrics were duration, angle, average angular velocity, starting angular velocity, middle angular velocity, ending angular velocity and maximum angular velocity of every turn. Start and end angular velocity were defined as the mean of the absolute value of the first or final 10 samples at the beginning or end of a turn, respectively. The middle angular velocity was defined as the mean of the absolute value of 10 samples at the middle of a turn. For the home environment data, the original definition of a turn (0.1–10 s duration, >90°) (Pham et al., 2017) was slightly adapted to increase the probability of identifying “true” turns in our analysis. We removed turns with a duration <0.5 s and >5.0 s, with an average angular velocity <20°/s and >360°/s and with a maximum angular velocity <20°/s and >720°/s.

### Statistical Analysis

We used JMP 11.2 software for statistical analysis. According to Delbaere et al. (2010a), we divided the overall cohort into the following four groups based on absence or presence of FOF and negative or positive fall history: “the vigorous” (i.e., without FOF and with negative fall history), “the anxious” (with FOF and negative fall history), “the stoic” (without FOF and with positive fall history) and “the aware” (with FOF and positive fall history). Demographic and clinical inter-group differences were calculated using ANOVA and *post hoc* Student’s *t*-test, except for gender, H&Y, freezing of gait, and CSF-amyloid-beta_1-42_, which were analyzed using chi-squared test. Shapiro–Wilk *W* test was used to test normality of data, and in case of non-normally distributed data, calculations were performed with the Kruskal–Wallis and *post hoc* Wilcoxon test. The results are presented as the mean and standard deviation, median and range, or proportion of the total. Due to the known disease severity and gender effects on turns (Haaxma et al., 2007), we evaluated the quantitative turning data with a linear regression model corrected for UPDRS III and gender. We did not include further parameters that could potentially influence turning metrics (such as LEDD, cerebrospinal fluid amyloid-beta_1-42_ level, or presence and severity of freezing of gait) in the model, as they did not significantly differ between groups (see below). The results are presented as the least squares mean and standard deviation. All *post hoc* analyses were Bonferroni corrected (0.05/4) since we performed four comparisons across groups that were considered relevant for the actual research question: anxious against vigorous and aware against stoic to evaluate the effect of FOF; stoic against vigorous and aware against anxious to evaluate the effect of fall history. For comparison between the lab and home assessments, we divided the home-based parameters by the respective lab-based parameters in those 28 patients who provided both home and lab data (**Table 3**). For this purpose, we used the means of both iTUG turns. This combination reflects, in our view, best the home situation.

## Results

In the lab-based substudy, we found that aware PD patients had a significantly longer least squares mean of turning durations during both turns of the iTUG compared to the stoic group (first turn, *p* = 0.002; second turn, *p* = 0.001). Accordingly, during the first turn, the middle angular velocity was lower in the aware PD patients than in the stoic ones (*p* = 0.04). During the second turn, the maximum angular velocity was lower in the anxious PD patients than in the vigorous PD patients (*p* = 0.04), and also lower in the aware PD patients than in the stoic patients (*p* = 0.014). We did not observe relevant differences between patient groups with and without positive fall history. Details are presented in **Table 1**.

From the home-based substudy, a mean of 9,664 turns per participant were included in the analysis. The only differences among all the quantitative turning parameters that remained significant after *post hoc* Bonferroni correction were the maximum and average angular velocities between the aware and stoic PD patients (*p* = 0.008 and *p* = 0.01, respectively, with lower values in the stoic PD patients). **Table 2** provides details regarding the turning metrics in the home environment.

**FIGURE 1:**
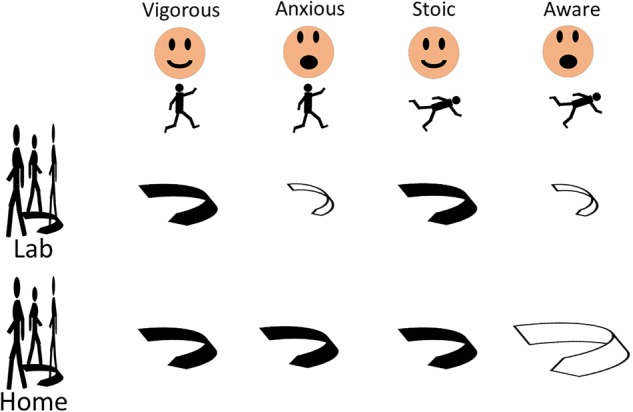
Fear of falling, but not a positive history of falls, induces changes of turning parameters in patients with Parkinson’s disease, regardless of history of previous falls and whether the turns are evaluated in the lab or the home. Significant differences compared to respective other groups (black arrows) are displayed with open arrows. Larger size of the open arrow indicates changes of turning parameters in direction “faster, more dynamic turn,” smaller size in direction “slower, less dynamic turn.”

The ratios between the home-based and the lab-based maximum and middle angular velocities were significantly different between the stoic and the aware PD patients (*p* = 0.005 and *p* = 0.004, respectively). The stoic PD patients turned at home approximately half as fast as during the lab assessment (ratio of home/lab = 0.60), whereas the aware patients showed virtually similar turning velocities under observed and unobserved conditions (ratio of home/lab = 0.95, **Table 3**). **Figure 1** provides an overview of the results.

## Discussion

To the best of our knowledge, this study is the first to focus on quantitative turning parameters in relation to FOF and a history of falls in PD patients. It shows that (1) FOF changes turning parameters, regardless of history of previous falls and (2) assessments should combine home and lab data, since they seem to bring different and potentially complementary information (**Figure 1**).

### FOF Changes Turning Parameters in PD Patients

Fear of falling does not only arise from previous falls; it is associated with the progression of the disease’s severity, a perceived imbalance based on dopaminergic medication and could be a psychological response to the diagnosis of PD (e.g., caused by near fall experiences and freezing of gait) (Bryant et al., 2014; de Souza et al., 2015). Several studies have reported that FOF is a substantial predictor of future falls, thus emphasizing the relevance of considering FOF for fall risk assessment in PD patients (Friedman et al., 2002; Pickering et al., 2007; Mak and Pang, 2009; Bryant et al., 2014). The situation is similar for the factor of a “positive history of falls” (Ashburn et al., 2001; Bloem et al., 2001; Almeida et al., 2015). The aim of the study was thus to determine whether FOF and a positive history of falls influence the quantitative turning parameters in PD based on previous assumptions that turning performance may be closely linked to fall occurrence and frequency (Cheng et al., 2014; Mellone et al., 2016; Yang et al., 2016; Fasano et al., 2017). Our results obtained in a lab environment demonstrate that aware PD patients turn slower than vigorous PD patients. This is in line with previous studies (Mak and Pang, 2009; Delbaere et al., 2010a; Bryant et al., 2014). However, this comparison does not allow a separation between the specific effects of FOF and a history of falls during turning. We therefore used a recently introduced approach for investigating these distinct associations in more detail (Pham et al., 2017). With this approach, we could determine that FOF – but not a positive falls history – has a relevant impact on the turning metrics in PD insofar as it was associated with obviously less stable turning behavior. In more detail, among the fallers, the aware PD patients (i.e., those with FOF) showed longer turn durations and lower middle angular velocities of turns, compared to the stoic patients (i.e., those without FOF). A trend toward the same direction was observable among the non-fallers. These results are supported by results obtained from the comparison of anxious (i.e., those without history of falls but with FOF) with stoic (i.e., those with history of falls but without FOF) PD patients. The anxious patients showed a longer turn duration and a lower maximum and middle angular velocity. As disease-associated factors and measures, such as MMSE, FOG, disease duration, LDEE and amyloid-beta values were not different across groups, and we corrected for motor impairment (i.e., MDS-UPDRS III), we suggest that our finding is not explained by these factors. Moreover, our results are in agreement with outcomes described in previous prospective studies showing that FOF has a negative impact on gait and balance in PD patients, regardless of previous falls (Rahman et al., 2011; Bryant et al., 2014). Due to FOF, PD patients have been shown in these studies to change gait speed, postural stability and general freedom of movement and action (Adkin et al., 2003; Mak and Pang, 2009; Lindholm et al., 2014). Future studies should evaluate whether selective interventions addressing FOF can lower fall risk and the number of falls in PD, specifically through an influence on turning performance.

### Different and Potentially Complementary Information From Lab and Home Assessment

Moreover, we were interested in whether an extensive assessment performed at home shows different results from the lab-based assessment, as there is increasing evidence that these two assessment strategies show substantially different, and at least partly complementary results (Fasano et al., 2017). In the home-collected data, we found partly opposing information to the data obtained in the lab. The stoic PD patients who showed a shorter turn duration and a higher angular turn velocity in the supervised (lab) environment compared to the aware PD patients presented with a lower average and maximum angular turn velocity in the home environment. We hypothesize that this effect is best explained by the use of different strategies during supervised movements (reflecting mostly physical capacity) and unsupervised movements (reflecting mostly physical activity). Our hypothesis is built on the following two observations: (i) physical capacity and physical activity domains do not highly correlate (Van Lummel et al., 2015; Giannouli et al., 2016; Zhang et al., 2017) and (ii) there is evidence of an increased activation of central compensatory networks in prodromal PD and clinical PD to address (involuntary, potentially reflecting “physical activity”-associated) motor deficits. (Buhmann et al., 2005; van Nuenen et al., 2012). Therefore, there may exist at least a subtype of patients that forces the affected patients to use the “physical capacity” mode instead of the “physical activity” mode for the successful performance of daily activities. Conversely, stoic PD patients demonstrated good physical capacity in the lab performance but obviously adapted their turning expenditures in the home environment to an extent that is less energy consuming. As our study design does not allow us to test for interaction effects and causal relationships, our interpretations need to be further investigated in future prospective and longitudinal studies that focus on a better understanding of axial impairment and milestones such as falls in PD (Puschmann et al., 2015).

### Positive Fall History Does Not Relevantly Influence Turning Parameters

Interestingly, a positive fall history did not contribute to an altered turning performance in our cohort. Although this lack of significant association might be explained by the relatively small sample size, the general assumption is that FOF has a more relevant influence on turning measures than a positive fall history in PD patients, both in the lab and at home. This result should motivate to a broad assessment strategy, including FOF aspects, in future studies focusing on mobility in this patient cohort.

As a general comment, most of our PD patients had relatively low H&Y stages, and the inclusion of PD patients with more advanced disease stages might have led to different results. Still, our study suggests that early treatment of FOF during the disease may prevent from future falls in at least a subgroup of PD patients.

## Conclusion

Our study suggests that FOF, much more than a positive fall history, has a relevant impact on turning metrics in PD patients under supervised and unsupervised conditions. It is thus probable that FOF contributes substantially to unsafe walking and can predict falls in PD patients. The opposing associations between FOF and the angular turn velocity measures in the supervised and unconstrained environments, as observed in the anxious and stoic PD patients, suggest that these two groups use different turning strategies during supervised and unsupervised assessment phases. Therefore, future studies in PD should include information from the lab in combination with data obtained from the home environment, as the latter data may be –at least partly– complementary to lab data.

## Author Contributions

LH and WM: research conceptualization, statistical analysis, drafting and revising the manuscript. LZ, SH, SN, MH, ME, CM, and LH: substantial contributions to the acquisition of data and organization of the study. LH, ME, MP, MH, and WM: analysis and interpretation of data. ME and WM: review of the statistical analysis. JvU, DB, and IL-S: contributed to the study organization and reviewing the manuscript. All authors revised the manuscript critically for important intellectual content and have given final approval of this version to be published.

## Conflict of Interest Statement

The recruitment of the cohort, including the collection of demographic and clinical data, has been funded by an unrestricted grant from Janssen Research and Development (Belgium), a pharmaceutical company of Johnson and Johnson. The analysis was partly supported by Lundbeck. This aspect does not alter the authors’ adherence to the journal’s policies on sharing data and materials. The authors declare that the research was conducted in the absence of any commercial or financial relationships that could be construed as a potential conflict of interest.
